# Microsensors for a Quick Detection of Tissue Functional Age and Precision Medicine

**DOI:** 10.33549/physiolres.935726

**Published:** 2026-06-01

**Authors:** Rossella SPERLONGANO, Michal VJACLOVSKÝ, Si CHEN, Martin MICHÁLEK, Lucie BLAŽKOVÁ, Aleksei PASHCHENKO, Leontýna VARVAŘOVSKÁ, Andrea FINDELAROVA, Bruno SOPKO, Taťána JAROŠÍKOVÁ, Alois NEČAS, Evžen AMLER

**Affiliations:** 1 Università della Campania Luigi Vanvitelli, Naples, Italy; 2 Second Faculty of Medicine, Charles University, Prague, Czech Republic; 3 FunGlass, Alexander Dubček University of Trenčín, Trenčín, Slovak Republic; 4 Department of Natural Sciences, Faculty of Biomedical Engineering, Czech Technical University in Prague, Kladno, Czech Republic; 5 Student Science s.r.o., Národních hrdinů, Prague, Czech Republic; 6 Faculty of Veterinary Medicine, University of Veterinary Sciences Brno, Brno, Czech Republic

**Keywords:** Biosensors, Nanofibers, Biomarkers, Precision medicine, Tissue aging

## Abstract

This proof-of-concept-study presents a novel microsensor based on specifically grafted functionalized glass for detection of low concentrated biomarkers in human fluids, gels, and for liquid biopsy. The device integrates functionalized nanofibers with grafted glass, creating an easy-to-use, rapid, and cost-effective biosensor. The approach combines fractionated nanofiber membranes with functionalized surfaces and light-scattering analysis on grafted glass which serves as a signal enhancer for data evaluation by light scattering. This method offers a novel tool for a quick evaluation of low concentrated protein samples. Experimental validation in this study confirmed its ability to identify low-abundance markers, supporting applications in precision medicine, tissue functional age assessment, and bacterial monitoring. Compared with conventional laboratory assays, optical sensors built on functionalized glass enable rapid, sensitive, and multiplexed biomarker detection directly at the point of care. We observed distinct changes in light transmittance caused by specific interactions between microsized particles and antibodies immobilized on the glass surface. This principle can also be applied to the direct detection of micrometer-scale systems such as bacteria, enabling fast urine analysis.

## Introduction

The demographic transition towards an aging population poses unprecedented challenges for healthcare systems worldwide. By 2030, more than one in five Europeans will be over 65 years old, with a corresponding rise in age-related diseases, frailty, and multimorbidity. This demographic shift has prompted major initiatives such as integration of molecular and physiological markers of aging into clinical practice to extend health span, reduce the burden of chronic disease, and support sustainable healthcare delivery. A quick and cheap determination of functional age in human fluids is attracting growing attention of modern geriatrics. Beside knowledge of appropriate markers for this determination, easy-to-use detectors are another most important assumption for assessment of functional age or for marker monitoring in precision medicine. Since these markers are usually low concentrated, filtration of human fluids seems to be an appropriate step towards a correct diagnosis since some human fluids, like urine, can be collected in larger volume and, thus, the markers can be concentrated after filtration.

Indeed, urine is an easily accessible human fluid which carries a rich cargo of metabolic byproducts, extracellular vesicles, proteins, and nucleic acids reflecting systemic physiological status. Knowledge of urinary biomarkers is particularly important. Indeed, calendar age usually does not match with functional age. Organs and tissues could become more or less used up in different individuals. Changes in urinary tract biology and molecular profiles reflect both calendar aging and emerging pathology and, by other words, also functional age. Elderly adults, particularly postmenopausal women, are more susceptible to recurrent urinary tract infections (UTIs) due to hormonal declines, weakened pelvic floor muscles, and impaired bladder emptying. Additionally, a healthy urinary microbiome undergoes shifts with age, influencing UTI risk and dysbiosis [5,6,7,8]. Traditionally, detecting pathogens like *E. coli, Enterococcus*, and *MRSA*, as well as dysbiosis in urine, requires cultures, which are time-consuming. However, new biosensor-based approaches offer rapid, point-of-care detection of uropathogens, potentially guiding antibiotic use in nursing homes and clinics. For instance, recent publications have highlighted the development of emerging nanostructured sensors for common uropathogens. Simultaneously, urine carries molecular signals of tissue stress and aging, including oxidative DNA damage (8-OHdG), profibrotic factors (TGF-β1 and matrix fragments), and senescence markers in urinary extracellular vesicles (e.g., p16^INK4a^). These biomarkers all increase with age or pathology. Unlike dipsticks, biosensors on functionalized glass could quantify these biomarkers cost-effectively, enabling routine screening of biological age and early disease [7,9,10,11,13,23]. Monitoring urinary profiles could thus provide a scalable approach for prescreening large populations. Beside knowledge of suitable markers, the easy-to-use detectors are another bottleneck for widely spread monitoring.

The aim of this manuscript is a proof-of-concept testing of microsensors based on specifically functionalized nanofibers for a quick, cheap and simple monitoring of an individual functional age. Clearly, the key element is the discovery and identification of cost-effective biomarkers that can stratify individuals by biological age and disease risk before overt clinical decline occurs. However, a cheap and quick monitoring technique is the second important step. We are introducing the next-generation glass-based optical nanosensors based on combination with fractionalized and specifically functionalized nanofibers. We take the advantage of nanofiber membranes which are characterized by an extremely large ratio between their surface and volume. Moreover, these membranes can be specifically functionalized on their surface by specific antibodies against markers which enables preparation of a highly specific and sensitive filters. These unique properties are combined with the unique grafted glass property: antibody against marker binding to the glass surface. This novel microsensor based on specifically grafted functionalized glass for detection of low concentrated biomarkers in human fluids, gels, and for liquid biopsy.

The approach combines fractionated nanofiber membranes with functionalized surfaces and light-scattering analysis on grafted glass and it serves as a signal enhancer for data evaluation by light scattering. This method offers a novel tool for a quick evaluation of low concentrated protein samples.

In fact, we suppose this sensor for a quick and cheap determination of low concentrated protein markers in human fluids in precision and personalized medicine, for determining tissue functional age as well as for monitoring of bacteria in human fluids or gels.

## Materials and Methods

### FTIR-ATR

Measurements of FTIR-ATR were performed on surface-primary-aminated microscope slides functionalized with (3-aminopropyl) triethoxysilane (APTES) kindly provided by University of Trencin, Slovakia). Qualitative and quantitative verification of surface modification was performed by Fourier Transform Infrared Spectrometer (IRAffinity-1S, Shimadzu Corporation, Japan) coupled with an Attenuated Total Reflection (ATR) accessory (Quest, Specac Ltd., UK). The infrared spectra were recorded in the 4000 to 400 cm^−1^ band, with 40 spectral scans acquired at a resolution of 4 cm^−1^. This technique is sensitive, relatively rapid, and suitable for routine assessment of surface amine functionalization [1,3,4].

### Colorimetric Orange II assay

The Orange II sodium salt colorimetric method was used to quantify the molar concentration of NH_2_ groups on functionalized surfaces. Add 50 μl of 5×10^−3^ M Acid Orange II (AOII, Sigma-Aldrich) HCl solution (1 mM, pH=3) to the surface of each sample. After incubation at 25 °C for 12 h, remove the supernatant from the plate and wash the samples with HCl solution (1 mM, pH=3) (3×15 min). Place samples in NaOH solution (0.01 M, pH 12) and incubate at 25 °C for 15 min to elute AOII adsorbed onto the sample surface. Finally, transfer 170 μl of eluate from each sample well to a 96-well plate and measure the absorbance value. Incubate for 15 min in NaOH solution (pH=12) to elute AOII adsorbed onto the sample surface. Finally, transfer 170 μl of eluate from each sample well to a 96-well plate and perform colorimetric absorbance measurements at 485 nm. The −NH_2_ concentration of each sample was determined by preparing an AOII standard curve at pH=12.

### Nanofiber preparation

Polyacrylonitrile (PAN) polymer (average molecular weight: 150,000 g·mol^−1^) was obtained from Sigma-Aldrich (St. Louis, MO, USA; product No. 181315). PAN nanofibers were supplied by Nanopharma (Czech Republic) and produced *via* an electrospinning process. According to the manufacturer, the spinning solution was prepared by dissolving PAN polymer in N,N-dimethylformamide (DMF) to obtain a homogeneous mixture suitable for electrospinning. The resulting nanofiber membrane had a basis weight of 17.3 ±0.6 g·m^−2^. Electrospinning parameters and processing conditions were optimized by Syndat to yield uniform, defect-free fibers.

### Nanofiber functionalization

After the preparation of PAN fibers, the surface modification was necessary. Surface modification – reduction – was performed to obtain suitable functional groups (−NH_3_) chemical bonds for subsequent functionalization, following the Draslovka a.s., Czech Republic, non-public internal procedure. PAN fibers were then functionalized (EBAS Nano, Czech Republic) by the covalent immobilization of anti-PSA antibodies kindly supplied by EBAS Nano. For each of the experiments the functionalization was made immediately before the experiment, in order to eliminate the storage/decay effect. The final concentration of the bonded antibodies was determined by infrared (IR) spectroscopy (IRAFfinity-1, Shimadzu, Japan) at 1750 s^−1^ and was estimated to be 106±16 μM g^−1^.

### Nanofiber fractionalization

Polyacrylonitrile (PAN) nanofiber mats were cut into small fragments using scissors and subsequently divided into two portions for processing. Fractionalization was carried out using a cryogenic grinder (Freezer/Mill 6870, SPEX SamplePrep, Metuchen, NJ, USA) equipped with small vials. Each grinding cycle consisted of 10 impact phases (1 min each) alternated with 1 min cooling intervals. The impact frequency during grinding was maintained at 10 Hz. Liquid nitrogen was used to ensure cryogenic conditions throughout the process, minimizing thermal degradation and preserving fiber integrity.

### SEM

**Fig. 1:**
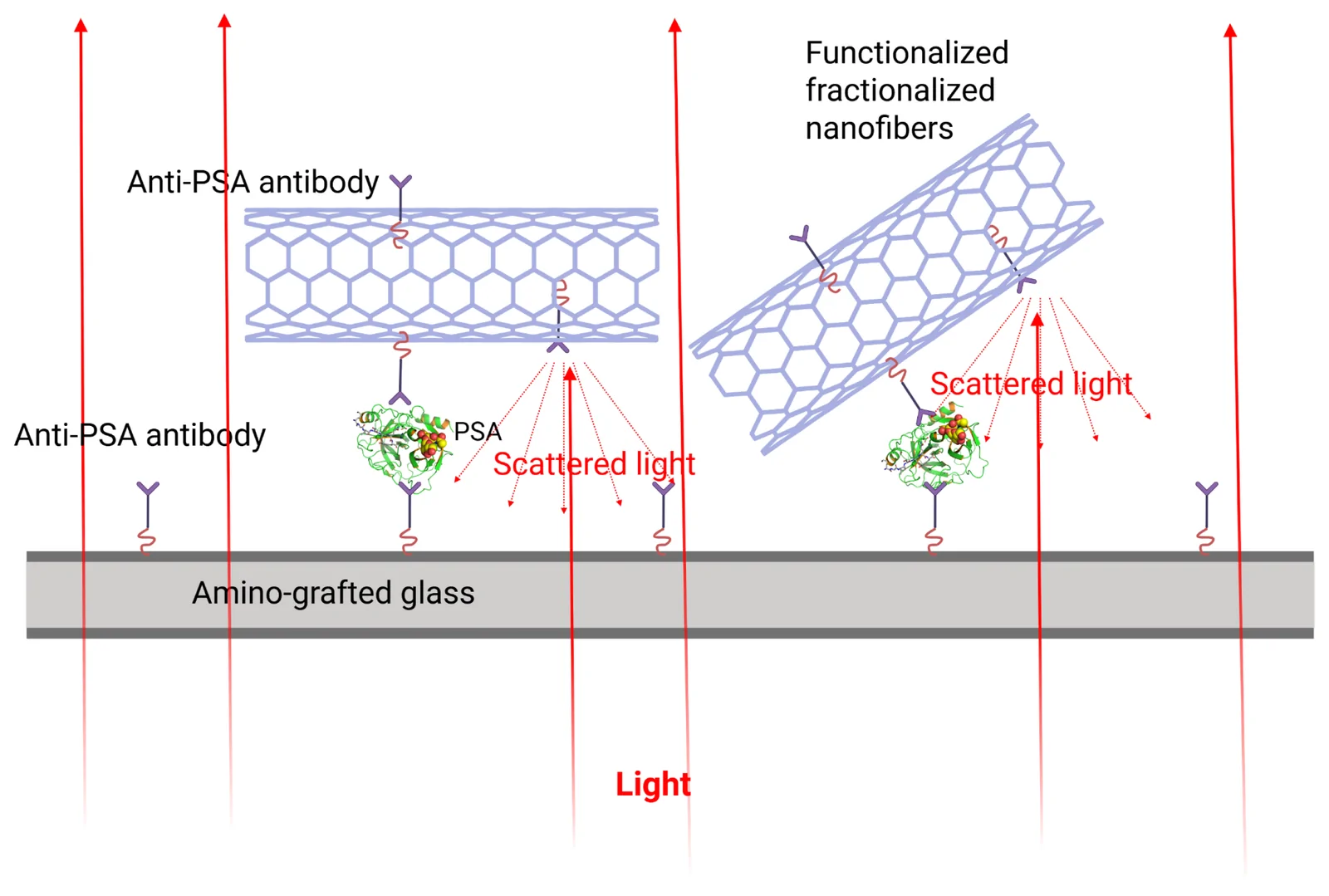
Concept of specific scattering on functionalized glass. The concept of the novel technology is based on a combination of fractionalized nanofibers functionalized with specific antibody against the marker bound on the smart glass surface. Fractionalized nanofibers are small enough to bind to the marker and, simultaneously, large enough for sufficient light scattering. Thus, this system could serve as an effective signal enhancer and can be used for monitoring of low concentrated markers by measurement of optical density.

The morphology of fractionalized PAN nanofibers was examined using scanning electron microscopy (SEM). Prior to imaging, samples were sputter-coated with a thin layer of gold using a rotary pumped coater (Q150R S, Quorum, Laughton, East Sussex, UK). SEM imaging was performed with a VEGA 3 SBU instrument (Tescan, Brno, Czech Republic) at magnifications ranging from 500× to 2000×, employing a secondary electron (SE) detector at an accelerating voltage of 10 kV. Fiber diameter measurements were conducted on 50 randomly selected fibers using Tescan software, and the average values were calculated to characterize the fiber size distribution (Fig. 1a).

### Transmittance measurement

The interaction between the marker-functionalized nanofiber membrane and the polyclonal antibody immobilized on the grafted glass was tested through the measurement of the transmittance. In general, glass is transparent for the whole spectrum of visible light. When particles set or bind to the glass surface, the light transmittance of glass decreases. To evaluate the mentioned interaction, the transmittance of the tested grafted glass was measured through the spectrophotometer Shimadzu UV-3600, Japan. First, samples of functionalized grafted glass were washed with distilled water to dispose of preservatives from the buffer (before use, the sample is stored in the PBS buffer with sodium azide at 4 °C. To obtain the transmission spectrum of the initial state, transmittance of individual glass samples was measured. Subsequently, the glass samples were exposed to the phosphate buffer solution (pH 7.4) with the fractionalized nanofibers and incubated on a rocking plate for 10 min at room temperature. After the incubation, the unbound particles were washed away with distilled water from the functionalized glass and the final transmittance was measured. The transmittance measurement was performed not only in the spectra with wavelength range of 180 to 700 nm, but also as a single point measurement at the wavelength of 530 nm.

## Results

### Concept and characterization of functionalized grafted glass

Our concept of a quick specific detection of small molecules in fluids (Fig. 1) is based on two main steps: first, filtration (or incubation) of fractionalized nanofiber membrane functionalized with monoclonal antibody against the marker on its surface and, second, secondary interaction of the marker on the nanofiber membrane with the polyclonal antibody on the grafted glass. The secondary interaction of the marker on the nanofiber membrane with a polyclonal antibody result in specific binding of microparticles on the surface of grafted glass. Presence of microparticles (with a size about one order larger than the wavelength of probing light) causes observable scattering even for low concentrated markers. Transparent grafted glass could, therefore, be subsequently used for a simple quantification by measurement of transmittance which decreases with increasing light scattering due to binding of microscopic particles.

This concept was experimentally proved. We have used surface-primary-aminated microscope slides functionalized with (3-aminopropyl)triethoxysilane (APTES) for preparation of our grafted glass (kindly provided by University of Trencin). The number of free amino groups was determined by a Fourier Transform Infrared as described in Methods and coupled with an Attenuated Total Reflection (ATR) accessory). The infrared spectra were recorded in the 4000 to 400 cm^−1^ band, with 40 spectral scans acquired at a resolution of 4 cm^−1^. Each sample underwent three FTIR-ATR measurements to verify reproducibility. The FTIR-ATR spectra revealed characteristic NH_2_-related vibration peaks, which serve as a fingerprint to confirm the successful grafting of amine groups onto the glass surface. These peaks typically include N-H stretching vibrations and out-of-plane N-H bending, with distinct shifts and new peaks compared to non-functionalized samples. By comparing spectra from unfunctionalized glass, NH_2_-RT (room temperature processed), and NH_2_-65 °C samples, changes related to the degree of primary amine functionalization can be identified [1,2].

**Fig. 2:**
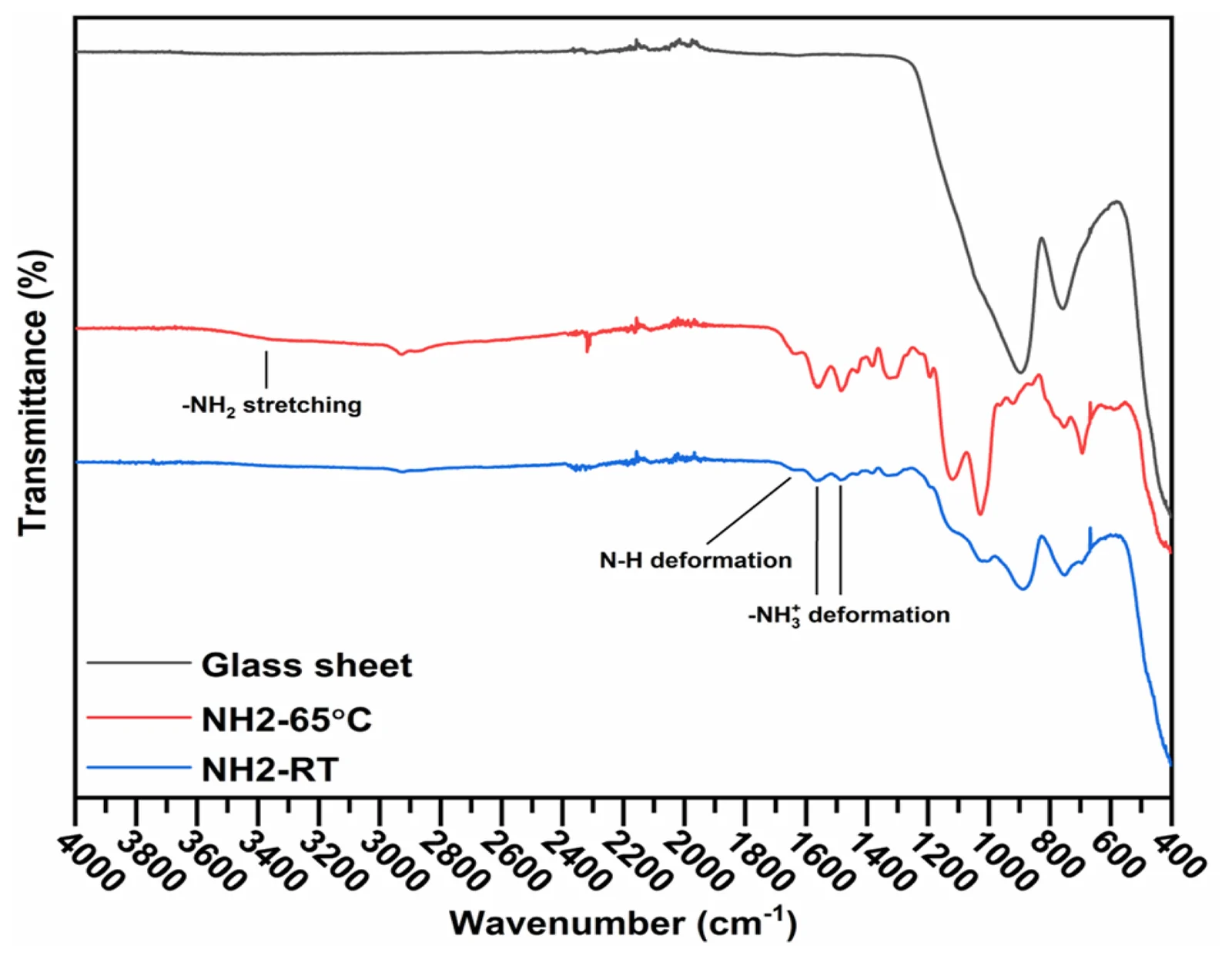
FTIR-ATR spectra. The FTIR-ATR spectra clearly show the NH_2_-related vibration peaks on grafted glass prepared under different temperatures (room temperature RT and 65 °C, respectively).

**Fig. 3:**
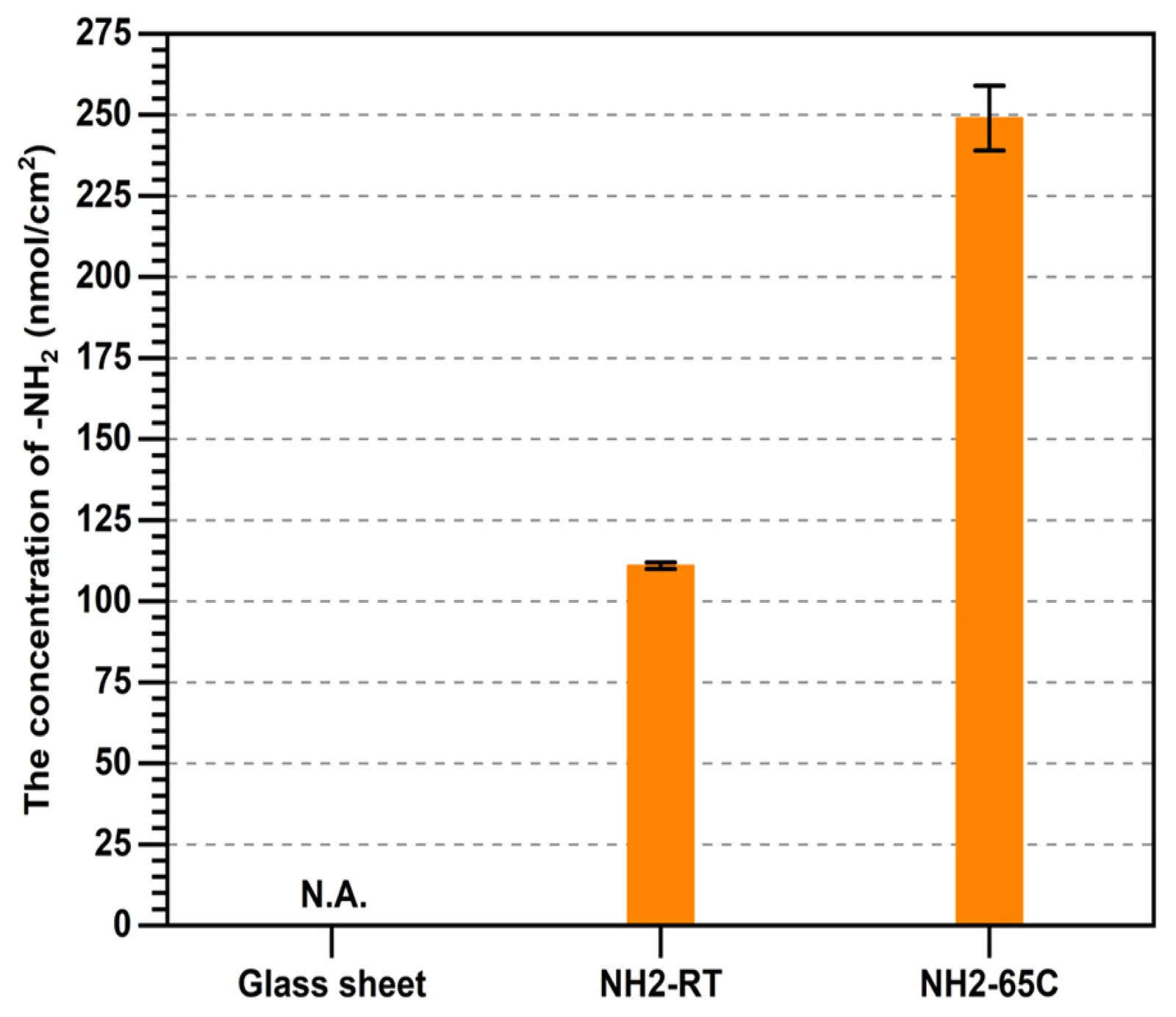
Temperature dependence of primary amine surface glass functionalization. Colorimetric Orange II Assay has been used for quantification of grafted amine groups on the glass surface. The induced NH_2_ concentration increased from less than 1 nmol/cm^2^ (estimated from the measurement error) prior the treatment by more than two orders and was found dependent on incubation temperature.

The FTIR-ATR spectra revealed characteristic NH_2_-related vibration peaks, which serve as a fingerprint to confirm the successful grafting of amine groups onto the glass surface (Fig. 2). These peaks typically include N-H stretching vibrations and out-of-plane N-H bending, with distinct shifts and new peaks compared to non-functionalized samples. The FTIR-ATR spectra clearly show the NH_2_-related vibration peaks on grafted glass prepared under different temperatures (room temperature RT and 65 °C, respectively). The induced NH_2_ concentration is dependent on incubation temperature. By comparing spectra from unfunctionalized glass, NH_2_-RT (room temperature processed), and NH_2_-65 °C samples, changes related to the degree of primary amine functionalization can be identified (Figs 2,3).

### Assembly of a specific microsensor and its testing

**Fig. 4:**
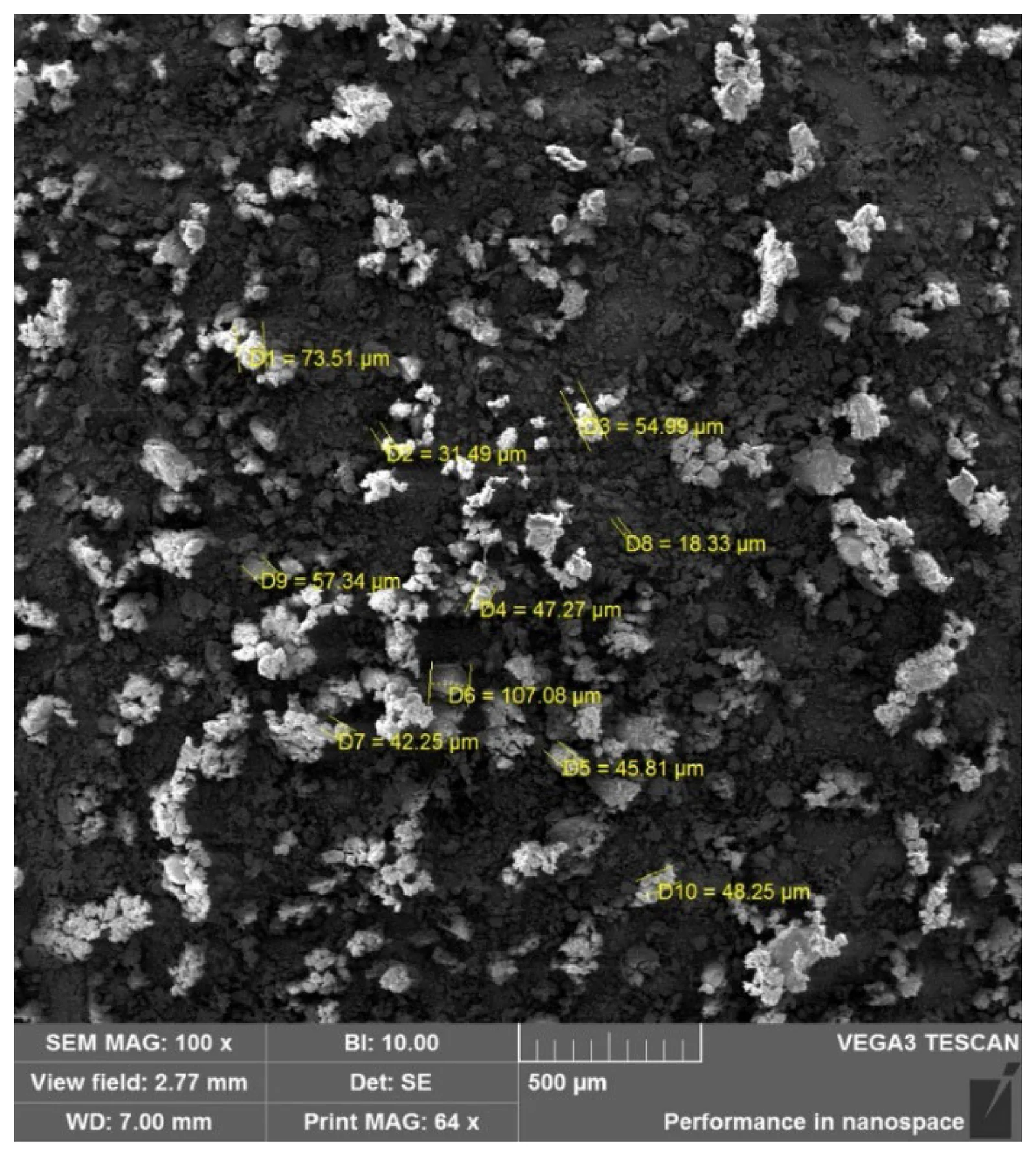
SEM image of fractionalized nanofibers. SEM photography of fractionalized nanofibers pressed into the filter for trapping of markers in fluid. The diameter distribution of fractionalized nanofibers used for specific interaction with the grafted glass surface is depicted as an inset in Fig. 2.

**Fig. 5:**
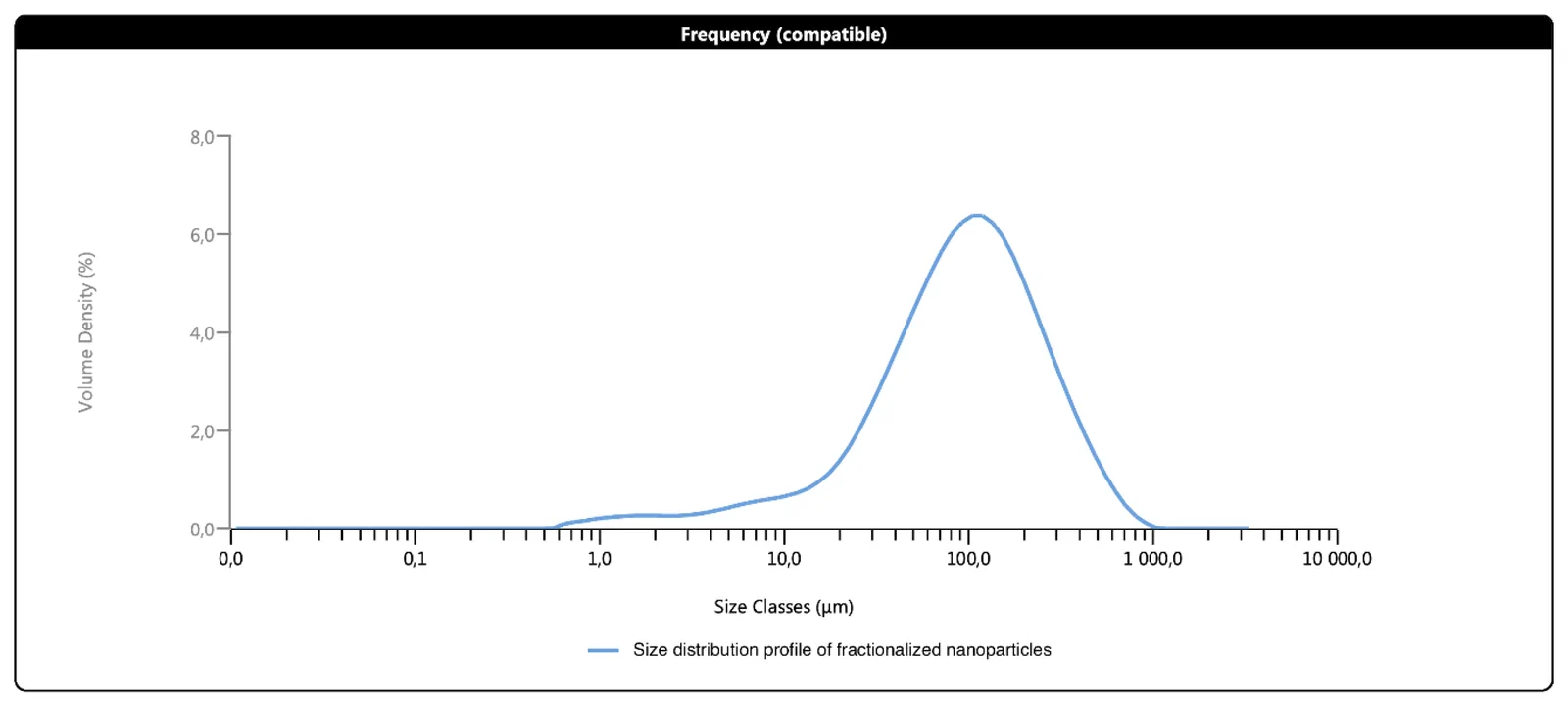
Size distribution of fractionalized nanofibers. Size distribution profile of particles obtained after cryogenic grinding of PAN nanofiber membrane.

First, the PAN nanofibers were reduced for further functionalization with a monoclonal antibody against PSA. To form microparticles and to avoid any structural collapse, nanofiber membranes were fractionalized at −196 °C. Cryogenic grinding resulted in production of microparticles with a preserved nanofiber substructure (Fig. 4). Diameters of fractionalized particles obtained from cryogenic grinding were analyzed by laser beam scattering on Mastersizer 3000 (Malvern Panalytical) and the mean value of the particle size is presented in Figure 5. Clearly, the size of particles is far above half of the visible light wavelength. Consequently, these fractionalized particles suitable for effective visible light scattering. Such a system kept all advantages of nanofiber binding sensitivity within microscopic particles that can assure pronounced light scattering.

Second, a similar approach was applied on the amino-grafted glass which was prior binding of the polyclonal antiPSA antibody further functionalized with a short linker using a crosslinking agent (kindly provided by ProNanoTech, s.r.o.).

The ability of fractionalized nanofibers to serve as an enhancer of light scattering was tested by PSA trapped on PAN nanofibers. Microfractionalized PAN nanofibers functionalized on their surface with antiPSA antibody were used as a filter for detection of PSA in model fluid. Since the nanofiber membrane is characterized with an extremely large ratio between surface and volume, the nanofiber membrane specifically functionalized with PSA antibody which we selected as a model marker, can bind the extremely low concentrated markers. We filtered 10 nM PSA in phosphate buffer, pH 7.4 through nanofiber filter. Filtrate was collected and filtration has been repeated through the same filter three times. Subsequently, the nanofiber filter has been thoroughly washed in a 10 mM phosphate buffer, pH 7.4 using gentle centrifugation for sample sedimentation. Grafted glass has been incubated with the sample fractionalized nanofibers as dispersion in 1 ml phosphate buffer, pH 7.4 on rocking plate for 10 min at room temperature. Incubated grafted glass was removed and washed and transmittance has been measured at 530 nm. As controls have been taken functionalized grafted glass without any treatment and also the glass without functionalization but treated as the sample. The experiment has been repeated three times.

Transmission of the sample (grafted glass after incubation) decreased about 50 % with respect towards the control (Table 1). This lower transmitted intensity clearly reflects an increased scattering due to specific binding of microparticles.

## Discussion

Our results have shown the viability of our proof-of-concept highlighting a novel approach in basic ageing biology, translational biomarker discovery, and device innovation. This and similar simple devices could have a high potential for development of next-generation screening tools for advanced personalized care for older adults. However, due to its simplicity and low cost, these devices can also mitigate escalating costs associated with late-stage disease management. The idea of human fluid filtration and gels analysis through fractionalized nanofiber membranes with specifically functionalized surface and subsequent monitoring of light scattering on grafted glass is a suitable approach and it has an ambition after optimization to serve for a quick monitoring of functional tissue age. Unlike conventional laboratory assays, optical sensors – particularly those built on functionalized grafted glass substrates – allow rapid, sensitive, and multiplexed detection of biomarkers at the point of care.

We demonstrated an easily detectable change in transmittance due to the specific interaction of micro-sized particles with antibodies on grafted glass. This approach can be overpassed for detection of micrometer scale systems, like bacteria, and these systems can be detected directly. Such a quick detector is highly desirable for urine quick analysis. Contrary to the long-held belief that urine is sterile, a diverse urinary microbiome exists even in healthy individuals. Moreover, age-related changes in this microbiota are now linked to UTIs, incontinence, and bladder cancer. For instance, studies have shown distinct urinary bacterial communities in pre- and postmenopausal women, indicating a hormonal regulation of urinary flora. However, simply carrying bacteria (bacteriuria) can remain asymptomatic until the immune system weakens [25].

Conventional UTI diagnosis methods, such as dipstick tests, microscopy, and culture, are too slow for prompt point-of-care decision-making. Consequently, advanced biosensors are being developed to rapidly identify uropathogens in urine, including *E. coli, Enterococcus, Staph aureus, Strep spp*., and resistance markers like ESBL. These biosensors utilize electrochemical and optical sensors functionalized with DNA probes or antibodies that can detect as few as 10^2^–10^3^ colony-forming units per ml, significantly lower than the typical culture thresholds. Application of grafted glass could serve as a quick and cheap alternative for rapid point-of-care detection in nursing homes and community clinics, these biosensors can facilitate immediate treatment and minimize complications. The application of grafted glass could serve as a quick and cost-effective alternative for rapid point-of-care detection in nursing homes and community clinics. These biosensors can facilitate immediate treatment and minimize complications.

Beyond infection, urine serves as a molecular readout of systemic oxidative stress, fibrosis, and cellular senescence – all hallmarks of aging. Non-invasive measurement of these biomarkers could reveal an individual’s biological age or early pathology even before symptoms manifest. Key candidate biomarkers include:

- Oxidative Stress: 8-hydroxy-2′-deoxygua-nosine (8-OHdG) reflects DNA oxidation. Large cohort studies indicate that urinary 8-OHdG (and related 8-oxoG) increases with chronological age and distinguishes individuals with accelerated aging. For instance, a study of 198 adults (age 20–89) showed a positive correlation between age and urinary 8-OHdG, with significantly higher levels in those with accelerated aging trajectories. Therefore, urinary 8-OHdG could signal organ stress and an increased risk of age-related diseases [26].
- Fibrosis Markers: Chronic organ overload often leads to fibrosis, such as kidney or liver scarring. Profibrotic cytokines like TGF-β1 and collagen/elastin breakdown products leak into urine during fibrotic remodeling. For example, urinary TGF-β1 levels correlate with tubulointerstitial fibrosis in kidney disease. Measuring such markers could reveal subclinical tissue damage. While not extensively studied in healthy aging, emerging data suggest that urinary extracellular matrix fragments and TGF-β1 increase with nephron loss and aging-related fibrosis.
- Senescence-Associated EVs: Senescent cells secrete distinctive extracellular vesicles (EVs) loaded with markers like the cyclin-dependent kinase inhibitor p16^INK4a. In human studies, urinary EVs from patients with hypertensive kidney injury carried higher levels of p16^INK4a, reflecting renal senescence. Generally, EVs are now recognized as “very important particles” in aging because they carry lipids, RNAs, and proteins that mirror a cell’s status, such as inflammation or DNA damage. Aptamer- or antibody-based assays on functionalized glass could selectively capture and quantify EV subpopulations (e.g., those bearing senescence markers), enabling a urine test for cellular aging [27].

Because urine can be collected noninvasively and in large volumes, combining multiple markers could yield a “biological age” score. For instance, a panel of oxidative stress markers (8-OHdG, protein carbonyls) and EV-based signals has been shown to predict age acceleration with high accuracy. In practice, these markers could serve as prescreening tools: a mild elevation might prompt lifestyle interventions, while larger deviations could trigger detailed imaging or laboratory workups for early organ pathology.

In addition, urinary biomarkers are already incorporated into screening protocols in various specialties across Europe and globally. By leveraging existing frameworks, new tests related to aging could be introduced as complementary tools. Examples include:

Prostate cancer: The urinary PCA3 assay (detecting prostate cancer antigen 3 mRNA) is FDA-approved to enhance risk stratification in men with elevated PSA [14,15,16]. Research has also identified newer urine proteins (SPON2, AMACR, TMEFF2, among others) that strongly correlate with prostate cancer grade. In a recent multi-cohort study utilizing AI on spatial transcriptomics, urinary SPON2, AMACR, and TMEFF2 were consistently overexpressed in high-grade tumors. Consequently, the resulting urine test achieved an AUC of approximately 0.92, significantly outperforming PSA [17,18,19,24].

## Expected Impact and Future Directions

A functionalized grafted-glass optical micro-sensor platform can enable rapid, non-invasive, and cost-effective detection of urinary biomarkers, with the potential to:

1. Assess functional (biological) tissue age through markers of oxidative stress, fibrosis, and cellular senescence.
2. Detect infections and other pathologies early, including uropathogens, at the point of care.
3. Support precision medicine in aging populations by allowing individualized risk stratification and timely intervention.

In essence, this technology links fundamental aging biology with practical, scalable biosensing, offering a realistic pathway toward preventive, decentralized diagnostics – particularly valuable in geriatric care and community settings.

Novel urinary biosensors aligns closely with the goals of geriatric care. Point-of-care optical sensors could enable the early detection of both infections and molecular signs of aging. For instance, a routine urine test could flag an impending urinary tract infection before the onset of fever, or rising oxidative stress could precede cognitive decline. This supports preventive interventions, such as antibiotics, hydration, or lifestyle changes, well before hospitalization becomes necessary. Similarly, integrating biological age assessment (*via* senescence markers) can inform personalized care plans. An “older” biological age might prompt more aggressive screening or rehabilitation, while a younger profile could reassure clinicians [22,25,26].

On a public health scale, incorporating urinary biomarker panels into general screening programs (e.g., for cancer or osteoporosis) would complement existing tests. For example, an annual health check could include a urine dipstick for uropathogens, along with assays for urinary tumor markers and bone turnover markers. The anticipated benefits include reduced invasive procedures (such as cystoscopy), improved therapy targeting (antibiotics, estrogen for pelvic floor, etc.), and more data to guide decisions. Over the next 5–7 years, small-scale clinical studies could validate candidate urinary markers in elderly cohorts, and pilot trials could test wearable or portable biosensor prototypes in nursing homes.

In summary, the integration of urinary biomarker research with optical biosensing technology holds transformative potential for geriatric diagnostics. It combines the fundamental concepts of cellular senescence and microbiome shifts with cutting-edge device engineering.

## Figures and Tables

**Table 1:** Transmittance of grafted glass before and after binding of microscopic particles.

Sample	Relative transmittance [%]
*Grafted glass without Ab (control 1)*	100 ± 1
*Grafted glass with Ab before binding (control 2)*	99 ± 1
*Grafted glass with Ab after binding*	49 ± 5
